# An Electromechanical Left Ventricular Wedge Model to Study the Effects of Deformation on Repolarization during Heart Failure

**DOI:** 10.1155/2015/465014

**Published:** 2015-10-15

**Authors:** B. M. Rocha, E. M. Toledo, L. P. S. Barra, R. Weber dos Santos

**Affiliations:** ^1^Graduate Program on Computational Modeling, Federal University of Juiz de Fora, 36036-900 Juiz de Fora, MG, Brazil; ^2^National Laboratory of Scientific Computing, 25651-075 Petrópolis, RJ, Brazil

## Abstract

Heart failure is a major and costly problem in public health, which, in certain cases, may lead to death. The failing heart undergo a series of electrical and structural changes that provide the underlying basis for disturbances like arrhythmias. Computer models of coupled electrical and mechanical activities of the heart can be used to advance our understanding of the complex feedback mechanisms involved. In this context, there is a lack of studies that consider heart failure remodeling using strongly coupled electromechanics. We present a strongly coupled electromechanical model to study the effects of deformation on a human left ventricle wedge considering normal and hypertrophic heart failure conditions. We demonstrate through a series of simulations that when a strongly coupled electromechanical model is used, deformation results in the thickening of the ventricular wall that in turn increases transmural dispersion of repolarization. These effects were analyzed in both normal and failing heart conditions. We also present transmural electrograms obtained from these simulations. Our results suggest that the waveform of electrograms, particularly the T-wave, is influenced by cardiac contraction on both normal and pathological conditions.

## 1. Introduction

The failing heart undergoes a series of changes, from electrophysiological alterations in ion channels, exchangers, and pumps to structural modifications of tissue properties, that provide the underlying basis for arrhythmias. Some notable characteristics of the failing heart include the prolonged action potential and alterations in the intracellular calcium handling, which alter the contractile function of myocytes [1]. This leads to a reduced ability of the left ventricle (LV) to efficiently pump blood and thus compromises normal heart function.

Electrophysiology in nonfailing (NF) and heart failure (HF) conditions is well described (see [2] for a review), but the coupled electromechanics of the heart is not. Cardiac contraction affects electrical activity of the heart through a series of complex interactions. For instance, at the myocyte level, the binding rate of Ca^2+^ to troponin-C depends on sarcomere length and some ion channels depend on the sarcomere stretch [3, 4]. At the tissue level, cardiac mechanics significantly contributes to the dynamics of complex reentrant waves [5] and also affects effective electrical tissue conductivities [6].

In [7], we presented a coupled electromechanical computer model of human left ventricle wedge preparation. This model was used to study how electrical activity triggers the contraction of the wedge and how its deformation affected repolarization and action potential duration (APD). We showed that, with deformation, the LV wedge stretches in the transmural direction, reduces the electrotonic effect, and thus increases the transmural dispersion of repolarization (TDR) and APD. These effects resulted in an increased T-wave amplitude on transmural electrograms computed from the simulations. This previous work has clearly showed a complex interaction between mechanics and electrophysiology.

In this work, we extended the strongly coupled electromechanical model used in [7] to represent HF changes at cellular and tissue level and then carried out simulations to analyze the effects of cardiac deformation on some important electrophysiology parameters. With this approach, using a left ventricular wedge* in silico* preparation, we investigated the effects of deformation on transmural dispersion of repolarization and action potential duration in NF and HF conditions. In addition, we also studied how deformation and HF influence the morphology of transmural electrograms obtained from the simulations of the LV wedge.

## 2. Physiological Models

To understand the effects of deformation on the transmural dispersion of repolarization in a normal and in a failing tissue we used a previously developed computer model of the human left ventricle wedge preparation [7, 10]. Here we present the models used to describe electrophysiology and mechanics. We also discuss how we modified our coupled electromechanical cell model for heart failure remodeling.

### 2.1. Cardiac Mechanics

Cardiac biomechanics was computed by solving the quasistatic equilibrium equations

(1) $$\begin{array}{c} \mathrm{div}\left(\;FS\;\right)=0, \end{array}$$

where **S** is the second Piola-Kirchhoff stress tensor. The second Piola-Kirchhoff stress tensor is twice the derivative of the strain energy function Ψ with respect to **C** = **F** ^*T*^ **F**, the left Cauchy-Green strain tensor. In this paper, a reduced transversally isotropic version of the orthotropic model of the cardiac tissue proposed by Holzapfel and Ogden (HO) [11] was used. Its strain energy function Ψ is given by

(2) ΨI1,I4f=a2bexp⁡bI1−3−1+af2bfexp⁡bfI4f−12−1,

where *a*, *a*_*f*_, *b*, and *b*_*f*_ are material parameters. This reduced version can be derived from the original model by simply setting *a*_*s*_ = *a*_*fs*_ = 0. We note that a similar approach was used in [12].

The fiber direction in the undeformed configuration is denoted here by **f**_0_. This version of the model has only 4 parameters and is defined in terms of the **C** tensor and the following invariants:

(3) I1C=tr⁡C,I4kC=k·Ck,I8fs=f0·Cs0,with  k=f0,s0.

The term containing the fiber invariant *I*_4*f*_ is not considered during compression, that is, when *I*_4*f*_ < 1, the contribution of the corresponding term to the strain energy function is neglected.

We used the active stress approach that splits the second Piola-Kirchhoff stress in passive and active stress parts. The passive part is given by the Holzapfel-Ogden model, described by (2), whereas the active stress contribution is given by

(4) $$\begin{array}{c} S^{a}=T_{a}^{\max}T_{a}\frac{f_{0}⊗f_{0}}{{\left|Ff_{0}\right|}^{2}}, \end{array}$$

where *T*_*a*_ is the normalized active force generated by the myocyte contraction model and *T*_*a*_^max⁡^ is a scaling factor to achieve the active stress found in cardiac myocytes [7].

### 2.2. Cardiac Electrophysiology

The electrophysiology of cardiac tissue, considering the effects of deformation, can be described by the bidomain model, which in this case is given by

(5) χCm∂Jv∂t+JIion−Div⁡JF−1DiF−TGradv=Div⁡JF−1DiF−TGradue,

(6) −Div⁡JF−1DF−TGradue=Div⁡JF−1DiF−TGradv,

(7) dsdt=gv,s,

where *v* is the transmembrane voltage, *u*_*e*_ is the extracellular potential, *I*_ion_ is the ion current of the cell model, *χ* is the surface to volume ratio, *C*_*m*_ is the membrane capacitance, **F** is the deformation gradient tensor, and *J* = det⁡(**F**). The conductivity tensor is defined as **D** = **D**_*i*_ + **D**_*e*_, where **D**_*i*_ is the intracellular tissue conductivity tensor and **D**_*e*_ is the extracellular tissue conductivity tensor. The function **g**, the components of the state variable vector **s**, and *I*_ion_ are determined by an ODE model of a cardiac cell, which will be described next.

Note that, in (5) and (6), the spatial derivatives are taken with respect to the original (undeformed) configuration, as described in [13]. Here, we used the modified bidomain model that takes into account the effects of deformation. In [7], we extended the monodomain model that considers deformation to this version of the bidomain model.

### 2.3. Human Ventricular Electromechanical Cell Model

Dynamics of human ventricular myocyte was described using a cell model that couples the electrophysiology model proposed by ten Tusscher et al. [14] and the myofilament model proposed by Rice et al. [15] for active force generation. Here this coupled electromechanical cell model will be referred to as TNNP + Rice model. A detailed description of the procedure used for coupling and model parameters can be found in [7, 16].

**Figure 1:**
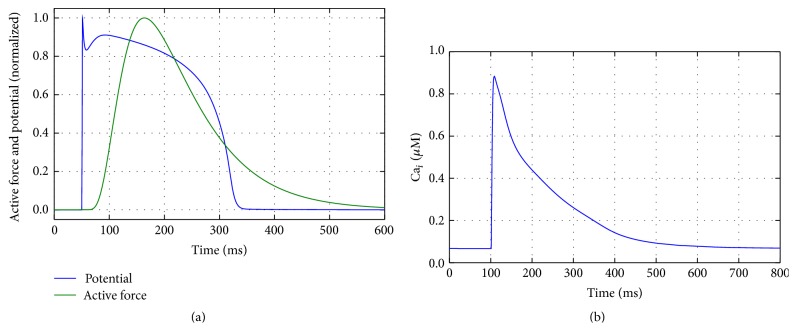
Coupled electromechanical TNNP + Rice cell model: (a) normalized transmembrane potential and active force and (b) intracellular calcium concentration.

The main variables of the TNNP + Rice cell model are the transmembrane potential, the intracellular calcium concentration Ca_*i*_, and the active force.  Figure 1(a) shows the normalized active force and transmembrane potential, whereas Figure 1(b) shows the intracellular calcium concentration of the coupled model.

### 2.4. Heart Failure Modeling at the Myocyte

Cardiac myocytes from failing hearts experience a series of changes; among them the most prominent changes are action potential prolongation and alterations in the intracellular calcium and sodium. Here we limit ourselves to describe the changes that were applied to our specific coupled electromechanical cell model; for more details about HF remodeling see [1, 17].

In this work, we modified the cell model by (i) adding the late sodium current, (ii) modifying some ion currents in a homogeneous way (same change for all cell types), and (iii) heterogeneous changes to ion currents and exchangers, that is, different changes for endocardial (endo), M, and epicardial (epi) cells.

The *I*_Na*L*_ current was modeled using the Hodgkin-Huxley formalism. Thus, as proposed in [17–19], the following equations were used for the late sodium current:

(8) INaL=gNa,L¯mL3hLv−ENa,

(9) dmLdt=αmL1−mL−βmLmL,

(10) dhLdt=hL,∞−hLτhL,

where *α*_*m*_*L*__, *β*_*m*_*L*__, *h*_*L*,*∞*_, and *τ*_*h*,*L*_ are given by

(11) αmL=0.32v+47.131−e−0.1+v+47.13,βmL=0.08e−v/11,hL,∞=11+ev+91/6.1,τh,L=233 ms.

Then, (9) and (10) were incorporated into the system of ODEs of the coupled TNNP + Rice model and the current *I*_Na*L*_, given by (8), was added to the total ion current *I*_ion_ of the model, which has the following form:

(12) Iion=INa+INaL+IK1+Ito+IKr+IKs+ICaL+INaCa+INaK+IpCa+IpK+IbCa+IbNa.

In [17], several modifications in the electrophysiological properties (ion currents and exchangers) are presented in order to describe heart failure using a human LV cell model. These modifications were incorporated in a homogeneous way (same change for endo, M, and epi cells) and are presented with respect to the original model in Table 1. A detailed discussion about the implications in the action potential profile of each change can be found on the original publication [17].

Table 2 presents the heterogeneous electrical remodeling of the failing cardiac myocytes incorporated into the coupled electromechanical cell model used in this work. NCX and SERCA denote the fluxes associated with Na^+^-Ca^2+^ exchanger and sarcoplasmic reticulum Ca^2+^ activity, respectively. The table also describes heterogeneous remodeling of the *I*_to_ and *I*_Ca*L*_ currents, as reported in [9]. We note that the changes in the *I*_Ca*L*_ current were slightly adapted from [9], whereas the other changes were applied with the values reported in [8, 9].

#### 2.4.1. Electrical and Mechanical Properties of the Failing Heart Tissue

During HF the cardiac tissue undergoes several changes in its properties; among them the changes in electrical conductivity and stiffness of the material are the most prominent. Experimental studies have shown alterations in Cx43 gap junctions, as well as a reduction in the conduction velocity of the electrical wave propagation in the cardiac tissue [20]. To account for these changes in our simulations, we reduced the values of electrical conductivity by 30%, based on values reported in [21].

To take into account the mechanical alterations in the failing heart, the constants of the HO constitutive model were modified as proposed in [21]: the model parameters were increased 5-fold. This resulted in an increase of the stiffness of the material, as observed in heart failure conditions.

## 3. Numerical Experiments

### 3.1. Numerical Methods

The electrophysiology models were spatially discretized using the finite element method (FEM) using trilinear hexahedral elements, whereas the time discretization was performed using the Crank-Nicolson method. For the bidomain system, we used the (*v*, *u*_*e*_) formulation which, after discretization, led to a system with a parabolic and an elliptic equations. We solved the bidomain equations using a decoupled approach where each equation is solved sequentially, one after the other. The preconditioned conjugate gradient method was used for the solution of the linear systems. For the elliptic part, a multigrid preconditioner was employed, whereas for the parabolic part an incomplete LU factorization was used as a preconditioner. The numerical integration of the system of ODEs that govern the dynamics of myocytes was performed using the standard Rush-Larsen method [22].

The numerical solution of cardiac biomechanics is more complex and requires a robust numerical treatment for incompressibility in order to avoid locking phenomena. In this work we used a mixed three-field variational formulation proposed by Simo et al. [23] for the solution of (1) using the HO constitutive model. The resulting nonlinear system of equations was solved using an incremental Newton-Raphson approach, which requires consistent linearization of all nonlinear involved quantities. More details about the numerical scheme for cardiac biomechanics and also electrophysiology can be found in [7, 10, 24] and the references therein.

The coupled electromechanical problem is solved sequentially using the same finite element mesh for both problems. Although the spatial scales of the electrical and mechanical problems are quite different, we adopted this approach due to its simplicity. First, we solve the bidomain model using an operator splitting approach: first, we advance the system of ODEs (TNNP + Rice model) at each node in time using the Rush-Larsen method [22] and then we solve the discretized parabolic and elliptic equations of the bidomain model. At every 1 ms of the coupled simulation, we compute the active stress (4) at the Gauss points and use it as the load in order to solve the equations of cardiac mechanics. Then, we obtain the deformation gradient tensor **F** which is used to introduce the effects of deformation in bidomain model (5) and (6) for the subsequent steps. More details about the numerical framework used for the solution of the electromechanical model can be found in [7, 24].

### 3.2. Simulation Setup

In this work, we considered simulations of the electromechanical activity of a left ventricle wedge under normal and heart failure conditions. The coupled electromechanical cell model TNNP + Rice was embedded in tissue simulations using bidomain and nonlinear elasticity equations. In both cases, we carried simulations without coupling the mechanics and with coupled mechanics to assess the effects of tissue deformation on electrophysiological metrics.

Under normal conditions, we considered a cubic domain of 9 × 9 × 9 mm^3^, whereas for the failing heart conditions we considered a slab of 13 × 9 × 9 mm^3^, both representing a transmural block of the human left ventricle. The transmural size of the LV wedge was based on data reported in [25]. The block was then subdivided into 3 regions to describe the endocardial, M, and epicardial regions. We used a distribution of 12% of endocardial cells, 60% of M-cells, and 28% of epicardial cells, since in a previous work [10] this distribution was able to reproduce a positive T-wave electrogram.

To simplify the simulations and the mechanical response, the behavior of the constitutive model and the conductivity tensor were assumed to be transversely isotropic. The fiber direction was assumed to be parallel to the *y*-axis, whereas the transmural direction was aligned with the *x*-axis. Since cardiac tissue contracts along the fiber direction, due to its incompressible behavior, we observe a stretch in the transmural direction, which is physiologically consistent.

An electrical stimulus was applied on the endocardial face of the mesh to initiate the electrical activity, which was simulated for *T* = 1000 ms. Boundary conditions for the mechanical equations were chosen in order to avoid rigid body motion and allow wall-thickening: the three faces contained in the planes *x* = *y* = *z* = 0 and the plane *z* = 9 mm for the NF case and *z* = 13 mm for the HF case were prescribed with zero normal displacement. For the electrophysiology part of the problem, no-flux boundary conditions were applied.

For each simulation, the activation time (ACT), repolarization time (REP), and action potential duration (APD) were computed for the nodes. Dispersion of ACT, REP, and APD were measured as the difference between the maximum and minimum values, that is, disp_APD_ = max⁡(APD) − min⁡(APD). Transmural electrograms were obtained using the bidomain simulations by computing the difference between the extracellular potential *u*_*e*_ from nodes positioned at the epicardial and endocardial surfaces.

In Table 3, an overview of the main parameters used for the simulations is presented. The values of electrical conductivities for the bidomain model were extracted from the literature [26]. The solution of the nonlinear elasticity problem used 10 load increments (*N*_inc_) and convergence was defined in terms of the energy norm. The values of the parameters of the HO constitutive model are reported in [27].

## 4. Results

### 4.1. Single Cell: Coupled Electromechanical Myocyte Model for Heart Failure

The results of incorporating the heart failure electrical remodeling changes, described in Tables 1 and 2, into the coupled electromechanical TNNP + Rice cell model are presented here.

**Figure 2:**
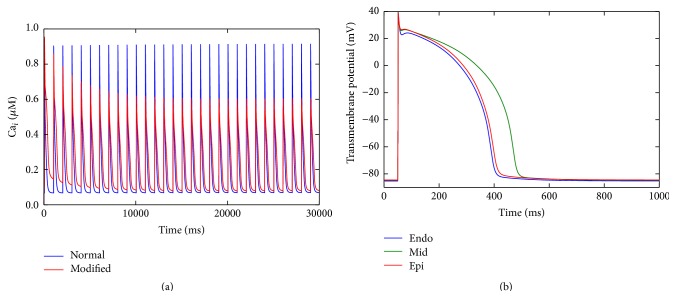
HF changes: (a) steady state calcium transient; (b) transmembrane potential for endo, M, and epi myocytes.

After the modifications for heart failure for each cell type (endo, M, and epi), a simulation was carried out applying an electrical stimulus with a frequency of 1 Hz until the model reached the steady state.  Figure 2(a) compares the calcium transient for the normal and modified (heart failure) cases. When the model reaches the steady state, all the values of its state variables are recorded and used as initial conditions. Note that, after the HF changes, the diastolic calcium increases and the peak of the calcium transient reduces significantly.

Figure 2(b) presents the action potential for the endo, M, and epi cells after the heart failure remodeling changes. A prolonged APD was obtained for all cell types; however, a greatly prolonged APD was observed for the M-cells, whereas for endo and epi cells only a modest prolongation was observed. Note that the APD of the endocardial cell was shorter than the epicardial cell, while in normal conditions the epicardial APD is shorter than the endocardial APD.

**Figure 3:**
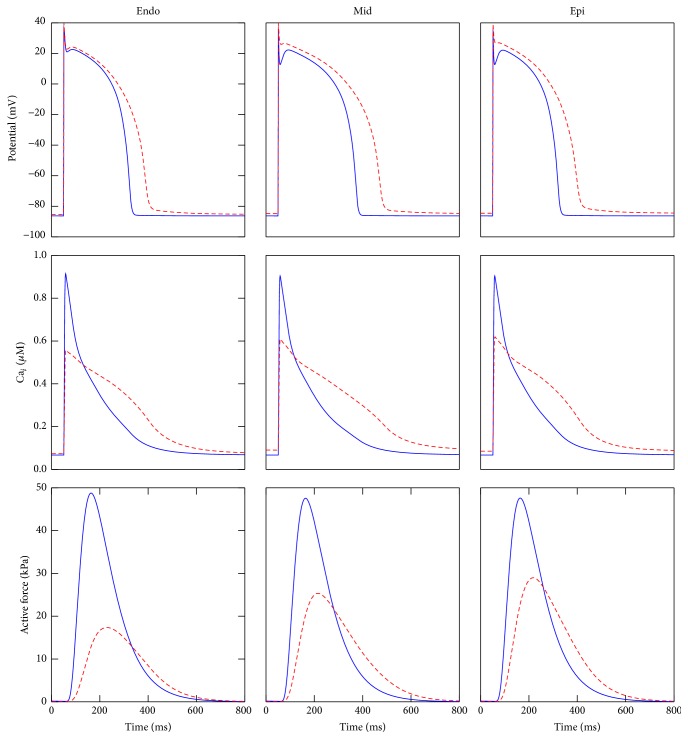
Action potential, calcium transient, and active force for cell types under normal (solid line) and failing heart (dashed line) conditions.

The results of the HF changes in the TNNP + Rice model in terms of transmembrane potential, intracellular calcium concentration, and active force are shown in Figure 3 for each cell type. As already mentioned, the prolongation of APD, which is a hallmark of failing heart [1], was observed for each cell type.

The intracellular calcium concentration in normal and HF conditions is shown in the middle of Figure 3. We observed that the peak was reduced by approximately 50% and also a reduction in the rate of decay when compared to the normal case was observed. In comparison with the normal case, the modified model was able to reproduce an important characteristic of HF which is the increase in the diastolic calcium.

In the coupled electromechanical TNNP + Rice cell model, the intracellular calcium concentration is used as input for generation of the active force, which is described by the Rice et al. model. Since, in the HF conditions, the intracellular calcium concentration changes, the resulting active force will change accordingly. The modified active forces for all the cell types are shown in the bottom of Figure 3. The changes were similar to the calcium transient, that is, a reduction in the peak and a longer duration of the active force. We note that the HF active force profile obtained here is consistent with other works [21, 28].

### 4.2. Control Wedge: Coupled Electromechanical Tissue Model

Before we discuss how deformation affects transmural dispersion of repolarization in HF conditions, we analyze first the interplay of mechanics and electrophysiology under normal or control condition. Thus, simulations of the cubic domain considering the transmural distribution of the cell types, with and without deformation, were carried out.

**Figure 4:**
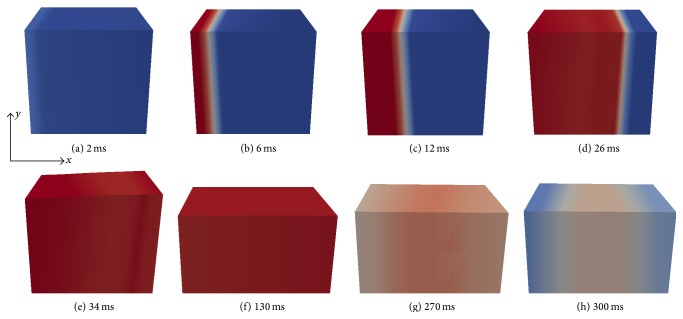
Spatial distribution of the transmembrane potential *v* in the coupled electromechanical simulation. Panels (e) to (h) show the transmural stretch of the LV wedge. *v* varies from −90 mV (blue) to 20 mV (red).

Figure 4 presents the results of the coupled electromechanics simulation (with deformation). The main mode of deformation is compression in the fiber direction and stretching in the transmural direction, that is, wall thickening. Note that, during the repolarization phase of the M-region, in Figures 4(g) and 4(h), the tissue is stretched.

The measures of ACT, REP, and APD are reported in Table 4. The dispersion of activation time is only slightly affected by deformation, since contraction starts when the tissue is already electrically activated. On the other hand, a significant increase in dispersion of repolarization time and action potential duration was observed between the simulations with and without deformation, with a difference of 10.7 ms and 6 ms for dispersion of REP and APD, respectively. We note that the profiles of ACT, REP, and APD obtained in this work, for both pure electrophysiology and electromechanics simulations, are similar to other works [29, 30].

**Figure 5:**
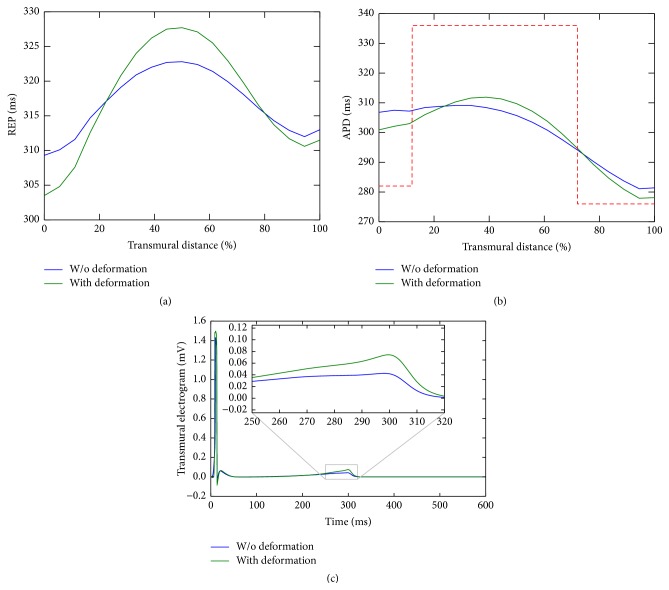
Comparisons of simulations with and without deformation (pure electrophysiology). (a) Repolarization time and (b) action potential duration in a transmural line of the domain. The dashed red line in (b) represents the single cell APD. (c) Simulated electrogram obtained with the extracellular potential *u*_*e*_ from the bidomain model.

Figure 5 shows a plot of the repolarization time and action potential duration taken from the the center of endocardial surface to the epicardial surface. It is clear that both REP and APD were affected by the deformation of the tissue, when compared to the simulation without deformation. This figure shows that, due to the stretch of the wall in the transmural direction, a reduction in the electrical coupling of the cells (or electrotonic effect) was observed, which was caused due to the fact that the wall thickening makes cells more distant from each other. In turn, this reduction in the electrotonic effect changed the APD of the cells such that they approached their intrinsic (single cell) APD, as shown in Figure 5(b) by the dashed red line.

The electrograms obtained from the extracellular potential *u*_*e*_, from simulations with and without deformation, are shown in Figure 5(c). Since depolarization is a fast phenomenon, the entire tissue is electrically activated before the onset of contraction. Therefore, as the figure shows, the QRS complex is not affected by mechanics. On the other hand, since the repolarization occurs after contraction and during the relaxation, the T-wave is affected by contraction, as shown by the inset in Figure 5(c).

An increase in the T-wave amplitude was observed in the coupled electromechanics simulation with respect to the simulation without deformation. The deformation causes the LV wall to stretch in the transmural direction, which reduces the electrotonic effect and therefore increases the transmural dispersion of repolarization, that results in an increase in the amplitude of the T-wave. This effect was studied previously in [7].

### 4.3. HF Wedge: Coupled Electromechanical Tissue Model

The coupled electromechanical model with heart failure is now studied using the same simulation setup already described but now considering the heart failure remodeling of the cell model and also the changes in tissue properties.

In the complete HF model, due to the reduced conductivity and increased size of the slab, we observed that the entire block of tissue is electrically activated after 36 ms, whereas in the normal case it took 25 ms to activate the tissue.

We observed that, in comparison with the normal case without heart failure, the LV wall thickening was reduced due to increased stiffness of the material and also due to the reduced active stress. In the simulations of the normal case, a maximum of 39% was obtained for the wall thickening, whereas in the hypertrophic heart failure case a maximum of 18% was achieved.

**Figure 6:**
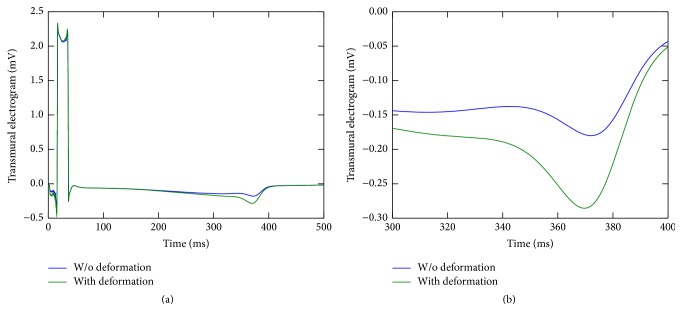
Transmural electrograms computed from simulations with and without (w/o) deformation for the HF case considering a hypertrophic LV wedge. On the right panel, the graph shows a zoom on the T-wave region.

Figure 6 shows the transmural electrogram computed from the results of the simulations using the complete HF model with a hypertrophic LV wedge. First, note that the electrogram is different from the normal case, shown in Figure 5(c), due to the negative T-wave. This occurred because, in the HF model, the APD of the endocardial cells is smaller than that of the epicardial cells and, therefore, they repolarize before the epicardial and M-cells.

As before, an increase in the T-wave amplitude (but now towards a more negative value) was observed in the simulations with deformation; see Figure 6(b). In the simulation without deformation a dispersion of REP of 46.2 ms was obtained, while in the simulation with deformation it increased to 55.7 ms, which means an increase of 6 ms. Again, the T-wave amplitude increase was associated with the increase in the transmural dispersion of repolarization.

It was shown in [7] that the more the LV wall stretches, the bigger the transmural dispersion of repolarization is. The increase in transmural dispersion of repolarization in turn affects the amplitude of the T-wave.

### 4.4. Discussion

#### 4.4.1. A Strongly Coupled Electromechanical Model of Heart Failure

Our HF wedge model that includes a strong couple between mechanics and electrophysiology was able to reproduce many known features of this particular pathology. Important changes in myocyte electromechanics were observed. Electrophysiological remodeling resulted in a prolongation of APD, in agreement with clinical and experimental observations [1]. In particular, the APD of the endocardial cell was shorter than the epicardial cell, while in normal conditions the epicardial APD is shorter than the endocardial APD. This is in agreement with the data and numerical experiments reported by [9] for dogs. However, in [9] the endocardial cell APD reduced when compared to normal case, whereas in the present work, we observed an increase of the endocardial APD in HF conditions when compared to the normal case, as suggested in other works [31].

In comparison with the normal case, the HF model was able to reproduce another important characteristic which is the increase in the diastolic calcium. It is important to note that the calcium concentration profile obtained in the simulation is similar to experimental [1, 9] and computational [8, 9, 17, 32] works. Since, in the HF conditions, the intracellular calcium concentration changes, the resulting active force changed accordingly; that is, the changes were similar to the calcium transient: a reduction in the peak and a longer duration of the active force. Once again, we note that the HF active force profile obtained here is consistent with other works [21, 28].

In the complete HF wedge model, LV wall thickening was reduced from 39% (control) to 18% (HF). Finally, from the computed transmural electrogram a negative T-wave was observed. This occurred because, in the HF model, the APD of the endocardial cells is smaller than that of the epicardial cells and, therefore, they repolarize before the epicardial and M-cells. This negative T-wave for the HF is in agreement with experimental, clinical, and computational works [9].

#### 4.4.2. The Impact of Tissue Stiffness

In the single cell simulations we have observed that both intracellular calcium and active force peaks were reduced in average by 50% (see Figure 3). Surprisingly, in the tissue model LV wall thickening decreased from 39% (control) to 18% (HF) (again 50%). Naively, one could interpret this results by linearly extrapolating single cell metrics to tissue ones. However, we highlight here that this is a coincidence and that the relation between the single cell active force and tissue contraction is nonlinear and very complex in general. First of all, we remind the reader that the result from the HF wedge simulation considered also that the tissue stiffness was greatly increased, a common feature of HF at its late stage. Therefore, both the decrease of myocyte's peak active force and increase of tissue stiffness contributed to the observed reduction of cardiac contraction in the HF wedge simulation.

**Figure 7:**
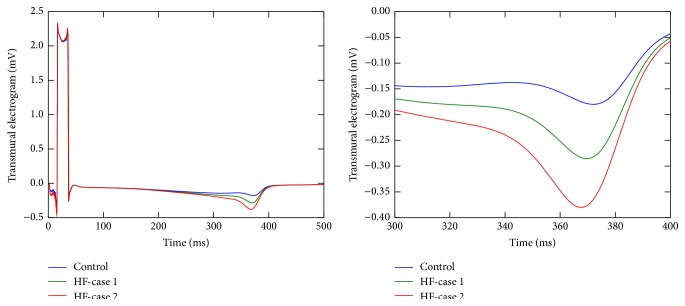
Comparison of the electrograms for the simulations considering HF conditions. Control denotes the HF simulation without deformation; HF-case  1 is the full HF remodeling with changes on both single cell and tissue properties; HF-case  2 is the HF remodeling case without changing the properties of the HO constitutive model.

To highlight how each of these changes influences cardiac contraction, we also simulated a second case of HF wedge (case  2) where tissue stiffness was unaltered; that is, the parameters that model it were set to the control ones. For this case, the LV wall stretched more than in the previous case, achieving 33% of wall thickening. Therefore, by only considering changes at the myocyte level, LV wall thickening decreased from 39% (control) to 33% (HF); that is, cardiac contraction was impaired but not as significantly as in the case that considers an increase of tissue stiffness. For completeness, Figure 7 shows the transmural electrogram for this case, including the electrograms computed for the previous cases for comparisons. The graphs clearly show the increase in the T-wave amplitude in both cases (1 and 2) with respect to the case without deformation. Here, it is clear that in case  2 (which considers normal values for tissue stiffness) the T-wave amplitude increased even more. These results can be explained again by the interplay of mechanics and APD dispersion.

#### 4.4.3. Information of Cardiac Mechanics in the T-Wave

It is clear from Figures 5(c), 6, and 7 that the repolarization phase of transmural electrograms is affected by cardiac contraction under normal and pathological conditions. Under normal conditions (see Figure 5(c)), the peak of normal T-wave is twice the one computed with a nondeforming wedge. For completeness, LV wall thickening was 39% and, as mentioned before, this dynamic contraction increased transmural APD dispersion by 6 ms, which resulted in the increase of T-wave peak.

Under the pathological condition of HF (see Figure 6), the peak of T-wave (which is now negative) is near twice the one computed with a nondeforming wedge. For completeness, LV wall thickening was 18% and this dynamic contraction also increased transmural APD dispersion by 6 ms (when compared to the same simulation without contraction).

Finally, under the pathological condition of HF without changes on tissue stiffness (case  2, see Figure 7), the peak of T-wave (which is negative) is near three times the one computed with a nondeforming wedge. For completeness, LV wall thickening was 33% and the dynamic contraction increased transmural APD dispersion by 10 ms (when compared to the same simulation without contraction).

Therefore, our results suggest that half of the information carried by the T-wave is somehow related to cardiac contraction. Both LV wall thickening and T-wave amplitude are metrics that can be clinically obtained in a noninvasive way by cardiac imaging techniques and electrocardiography, respectively. However, the relation between these two metrics is nonlinear and as the numbers above suggest, one can not make a straight forward relation between LV wall thickening and T-wave amplitude. T-wave peak can be more easily associated with APD or repolarization dispersion. But dispersion of repolarization is affected by myocyte electrophysiology, myocyte phenotype distributions, tissue properties, and contraction in a very nonlinear and complex fashion.

### 4.5. Limitations

#### 4.5.1. Apex-Base Heterogeneity

In this work the transmural heterogeneity of action potential duration of endocardial, M, and epicardial ventricular myocytes was considered. However, the apex-to-base gradient of action potential duration was neglected. Further studies should consider the apex-base gradient, which is an important electrophysiological characteristic of the left ventricle as demonstrated in [33].

#### 4.5.2. Wedge versus LV Geometry

Another strong limitation of this work consists in the fact that only a transmural slab of the LV was used in the simulations. Simulations of the entire left ventricle geometry, including both transmural and apex-base action potential duration gradient, are the next steps of this work.

The contraction of the LV is composed of three different mechanisms: circumferential shortening, longitudinal shortening, and wall thickening [34]. Circumferential shortening is the result of the contraction of the midwall fibers, while the longitudinal shortening is mainly caused by the contraction of oblique epi- and endocardial fibers. Wall thickening, however, is more complex and is both influenced by circumferential shortening and the interaction between oblique epi- and endocardial fibers.

Our current, simplified, wedge model achieved 39% of wall thickening in the NF case, which is very close to the physiological values which ranges between 40% and 50%, as reported in the literature [25]. However, it is important to note that the model we used is limited since the fiber distribution does not account the transmural rotation of the fiber and boundary conditions neglect the effect of blood pressure on the endocardium surface. In addition, the constitutive model reduces to an isotropic case due to the compression in fiber direction.

A wedge model with a physiological fiber distribution (transmural rotation of the fibers from endo- to epicardial surface) might also be able to reach the physiological range values of wall thickening of the LV. However, since WT is the combined result of a circumferential shortening and endo- and epicardial fiber contraction, it would probably provide smaller values for wall thickening.

It is important to remark that our focus was on the relationship between wall thickening and electrophysiological properties. Therefore, with this in mind, we decided to use a simplified fiber distribution for the wedge model which resulted in the moderate but physiological wall thickening of 39%, as mentioned before.

#### 4.5.3. Electrophysiology and Deformation

In general, each of the constitutive properties of the electrophysiological model (monodomain or bidomain) may depend on the state of deformation. For the monodomain model, this results in

(13) $$\begin{array}{c} \nabla \cdot \left(\;D\left(\;F\;\right)\nabla v\;\right)=\chi \left(\;F\;\right)\left(\;C_{m}\left(\;F\;\right)\frac{\partial v}{\partial t}+I_{\text{ion}}\left(\;F\;\right)\;\right), \end{array}$$

where **F** is the deformation gradient tensor. This equation shows that the electrical properties of cardiac tissue, namely, the membrane capacitance *C*_*m*_, the ionic current *I*_ion_, the conduction tensor **D**, and also the surface-to-volume ratio *χ*, depend on the deformation. The influence of deformation on *I*_ion_ is through the stretch-activated channels (SACs), which is a component of the total ionic current *I*_ion_.

In this work, we have neglected the capacitive, the ionic, and also the surface-to-volume ratio dependencies on deformation, as presented in [5]. Instead, we have simply focused on the effects of tissue deformation on diffusion, which is described by the first term of (13). To the best of our knowledge, the relationship between the surface-to-volume ratio and deformation in cardiac tissue has not been addressed so far. For details about stretch-activated channels see [35], whereas details about the capacitive dependency can be found in [36].

#### 4.5.4. Parallel Solver for Coupled Electromechanics

Our current implementation is limited and would not support an entire human left ventricle. To consider such geometry, we need to parallelize our code using a distributed memory approach with MPI. Another possibility, which has been shown to improve significantly the performance [37], is to exploit the computational power of modern GPUs to carry out the electromechanical simulations.

## 5. Conclusion

In this work, we presented a strongly coupled electromechanical model of a human left ventricle wedge preparation suitable for analyzing the effects of cardiac tissue deformation on electrophysiological metrics. We adapted our cell model to reproduce heart failure conditions and embedded this model in tissue simulations. Within this framework, we observed that also in HF conditions the deformation of the tissue reduces the electrotonic effect and consequently increases TDR and APD dispersion. The computed transmural electrograms presented a negative T-wave due to HF remodeling. Nevertheless, even in HF conditions with a negative T-wave, the wall thickening of the LV resulted in an increase of the T-wave amplitude.

Therefore, our results suggest that in both normal and HF conditions half of the information carried by the T-wave is related to cardiac contraction. Both LV wall thickening and T-wave amplitude are metrics that can be clinically obtained in a noninvasive way by cardiac imaging techniques and electrocardiography, respectively. However, we have shown that the relation between these two metrics is complex and nonlinear, which prevents a direct correlation of these two important clinical metrics. Only using a sophisticated and strongly coupled electromechanical model, we were able to correlate cardiac contraction and T-wave. This work highlights how important it is to further improve cardiac models so that they can be used as another important complementary tool in clinical cardiology.

## Figures and Tables

**Table 1:** Homogeneous heart failure remodeling in cardiac myocytes.

Current	Parameter	Change (%)
*I*_*K*1_	*g*_*K*1_	↓33
*I*_Na*K*_	*P*_Na*K*_	↓10
*I*_*b*Ca_	*g*_*b*Ca_	↑153
*I*_leak_	*V*_leak_	↑500
*I*_Na*L*_	*g*_Na*L*_	↑200

**Table 2:** Heterogeneous heart failure remodeling in cardiac myocytes.

	Parameter	Epi (%)	M (%)	Endo (%)	Reference
SERCA2a	*V*_maxup_	↓30	↓40	↓45	[8]
NCX	*K*_NaCa_	↑200	↑165	↑165	[8]
*I*_Ca*L*_	*g*_Ca,*L*_	↔	↓20	↓35	[9]
*I*_to_	*g*_to_	↓70	↓70	↔	[9]

**Table 3:** Parameters used in the numerical experiments.

Parameter	Value (unit)
Active stress	*T*_ref_^max^ = 50 kPa
Capacitance	*C*_*m*_ = 1.0 *μ*F/cm^2^
Surface-to-volume	*χ* = 2000 cm^−1^
Conductivities	*σ*_*i*_^*f*^ = 3.0 mS/cm, *σ*_*i*_^*s*^ = 1.0 mS/cm
	*σ*_*e*_^*f*^ = 2.0 mS/cm, *σ*_*e*_^*s*^ = 1.65 mS/cm

Discretization	Δ*x* = 500 *μ*m, Δ*t*_ode_ = Δ*t*_pde_ = 0.05 ms
	Δ*t*_mec_ = 1.0 ms
Solution	*N*_inc_ = 10, Δ*E*_*k*+1_ ≤ 10^−6^Δ*E*_1_

**Table 4:** Minimum (min), maximum (max), and dispersion (disp) of ACT, REP, and APD computed from the simulations with and without considering deformation.

	Without deformation			With deformation		
	min	max	disp	min	max	disp
ACT (ms)	2.5	31.6	29.1	2.6	33.4	30.8
REP (ms)	309.3	322.8	13.5	303.5	327.7	24.2
APD (ms)	281.1	309.1	28.0	277.9	311.9	34.0
